# Systemic and local antiinflammatory effect of magnesium chloride in experimental arthritis

**DOI:** 10.1186/s42358-023-00346-8

**Published:** 2024-01-04

**Authors:** Ana Carolina Matias Dinelly Pinto, Rodolfo de Melo Nunes, Waleska Vidal de Freitas Carvalho, Virgínia Claudia Carneiro Girão, Francisco Airton Castro Rocha

**Affiliations:** 1 Departamento de Medicina Interna da Faculdade de Medicina da Universidade Federal do Ceará, Fortaleza - Ceará, Brazil; 2 Programa de Pós-Graduação em Biotecnologia em Saúde Humana e Animal da Universidade Estadual do Ceará, Fortaleza - Ceará, Brazil; 3 Departamento de Morfologia da Faculdade de Medicina da Universidade Federal do Ceará, Fortaleza - Ceará, Brazil; 4 Instituto de Biomedicina – Laboratório de Investigação em Osteoartropatias, Rua Coronel Nunes de Melo, 1315 -1º. Andar Rodolfo Teofilo, Fortaleza, CE, CEP: 60430-270 Brazil

**Keywords:** Magnesium, Inflammation, Pain, Arthritis, Zymosan

## Abstract

**Objective:**

Despite some knowledge gaps in scientific evidence, MgCl_2_ is largely used for pain relief in musculoskeletal diseases. Mg salts were shown to provide analgesia postoperatively in orthopedic surgery and low Mg levels were linked to arthritis development and severity. We determined the anti-inflammatory activity of MgCl_2_ in an acute arthritis model.

**Methods:**

Mice received 0.1 mg/25µL Zymosan (Zy) or saline into the knees. Joint pain was evaluated using von Frey test; cell influx, and interleukin (IL)-1 level were assessed in joint lavage at 6 h. Synovia were excised for histopathology and analysis of immunoexpression of nuclear factor kappa B (NFκB) and tumor necrosis factor (TNF)-α. Groups (n = 6/group) received either 90 mg/kg MgCl_2_/100 µL or saline *per os* (systemic) or 500 µg/25 µL MgCl_2_ or saline intra-articularly (i.a.) 30 min prior to Zy.

**Results:**

MgCl_2_ given either systemically or locally significantly reduced cell influx (*p* = 0.0012 and *p* = 0.0269, respectively), pain (*p* = 0.0005 and *p* = 0.0038, respectively), and intra-articular IL-1 level (*p* = 0.0391), as compared to saline. Systemic MgCl_2_ significantly decreased NFκB (*p* < 0.05) immmunoexpression, as compared to saline.

**Conclusion:**

MgCl_2_ given systemically or locally displayed anti-inflammatory activity in a severe acute arthritis model reducing cell influx, pain, and cytokine release. MgCl_2_ operates at least partially via inhibiting NFκB activation. This is the first in vivo demonstration that MgCl_2_ decreases cytokine release in arthritis, prompting reduction of inflammation and pain relief.

## Background

Despite some knowledge gaps in scientific evidence, magnesium chloride (MgCl_2_) supplementation has become popular as a compound to treat osteoarthritis (OA). Curiously, magnesium sulfate (MgSO_4_) has been for long time used in anesthesia as a strategy to reduce the need for opioids postoperatively as well as a substance to facilitate anesthetic procedures including intubation. MgSO_4_ is also a first option to treat or prevent seizures linked to pre-eclampsia [1]. Notwithstanding, intra-articular injection of MgSO_4_ was shown to decrease pain in patients subjected to knee arthroscopy [2]. Magnesium is a micronutrient involved in the regulation of physiological functions in the body. It ranks fourth among cations present in extracellular fluids, being deposited in bones, teeth and soft tissues [3]. A recent study using an OA model has shown that MgCl_2_ given intra-articularly prevents progression of joint damage, which was associated to decreased expression of matrix metalloproteinase and interleukin-6 genes in synovium and cartilage explants, thus suggesting possible anti-inflammatory and cartilage sparing effects [4]. Also, lower serum magnesium levels, even within the normal range, were associated with higher prevalence of knee chondrocalcynosis, i.e. calcium pyrophosphate deposition disease (CPPD), in two large population based Chinese cohorts [5]. Moreover, using data from the Osteoarthritis Initiative cohort, lower serum magnesium levels were associated with worse pain and function in individuals with knee OA [6]. In keeping with these data, a narrative review approaching magnesium deficiency found association with development of diverse chronic inflammatory diseases, including atherosclerosis, diabetes, and rheumatoid arthritis. Another more recent review focus the various mechanisms involved in Mg role in inflammation [7, 8]. We are not aware of studies investigating anti-inflammatory efficacy of systemic, oral administration, of MgCl_2_ in arthritis models. Given that patients with arthritis usually ingest MgCl_2_ as a pain killer, our objective was to determine whether MgCl_2_ displays anti-inflammatory and antinociceptive activity after local and systemic administration in an acute arthritis model, while unraveling a possible mechanism of action.

## Methods

### Animals

Male Swiss mice weighing 20-25 g were obtained from the Central Animal Facility of the Faculty of Medicine of the Universidade Federal do Ceará, Brazil. All efforts were made to minimize suffering, with a total of 48 animals being housed in temperature-controlled rooms with 12 h light/dark cycles and free access to water and food. Careful monitoring and maintenance were conducted in accordance with the ethical recommendations of the Brazilian Veterinary Medicine Council (CMV), Guidelines for the Use of Animals in Research of the International Association for the Study of Pain (IASP), and the Guide for the Care and Use of Laboratory Animals (National Institutes of Health Publication). Additionally, protocols employed were approved by the local Ethics Committee on animal experimentation (Reg. Nº. 13,307).

### Zymosan arthritis model – assessment of cell influx and joint nociception

Mice received either intra-articular (i.a.) injection of 0.1 mg/25µL Zymosan (Zy; Sigma, St. Louis, MO) or saline (control) into the right knee joints under xylazine (10 mg/kg) / ketamine (80 mg/kg) intra-peritoneal (i.p.) anesthesia. After 6 h, mice were terminally anesthetized and the exudates of the knee joints were collected by aspiration for determination of total and differential cell counts using Neubauer chamber and Panoptic Instant Prov™ staining kit (New ProvBrasil™), respectively. Nociceptive behavior was assessed using the electronic pressure-meter nociception paw test by an observer blinded to group allocation. Animals were placed in acrylic cages (12 × 10 × 17 cm high) with a wire grid floor, 15 min before the beginning of the tests, in a quiet room. Stimulations were performed only when animals were quiet, without exploratory, urination or defecation movements and not resting on their paws. The electronic pressure-meter consists of a hand-held force transducer fitted with a polypropylene tip (Electronic von Frey aesthesiometer, Insight Equipamentos Científicos Ltda., Brasil). The polypropylene tip was applied perpendicularly to one of the five distal footpads of the right hind paw. The intensity of the stimulus was automatically recorded when the paw was withdrawn. The test was repeated three times, until less than a 1 g difference between measurements was obtained. Results were expressed as the mean value of three withdrawal threshold measurements (g).

### Pharmacological treatments

Groups (n = 6/group) received either 90 mg/kg MgCl_2_ or saline (100 µL total volume) *per os* (p.o.; gavage; systemic intervention) or 500 µg MgCl_2_ solution or saline (25 µL total volume) i.a. (local intervention) 30 min prior to Zy injection. The intraarticular dosage of MgCl_2_ was based on a previous study showing that 500 µg MgSO_4_ given intra-articularly to rats subjected to experimental OA reduced both pain and joint damage [9]. Oral dosage of Mg was based on a previous study showing that rats given 50-200 mg of Mg per liter in drinking water (drinking roughly 10mL/d) had less inflammation when subjected to experimental arthritis [10]. In that case, each rat would receive 0.5 -2 mg Mg daily. In the present study, Swiss mice received 90 mg/Kg MgCl_2_*per os* meaning each mice received a single daily dosage of 4 mg MgCl_2_ which would be equivalent to roughly 1 mg Mg daily.

### Synovial histopathology

Mice were sacrificed six hours after the intra-articular challenge with Zy and had their knee joint tissues excised for the histological study. After fixation in 10% v/v formaldehyde solution and decalcification (5% v/v formic acid in 10% v/v formaldehyde solution), the whole joint, comprising the distal femoral and proximal tibial extremities, was processed for paraffin-embedding and staining with hematoxylin-eosin (HE). Analysis was expressed as one result/ sample. Semi-quantitative histopathological evaluations were performed by an independent observer (VCCG) blinded to group allocation considering synovial proliferation and cell infiltration, ranging from 0 to 3 (0, absent; 1, mild; 2, moderate; 3, severe). Results are expressed as the median value for each group of at least four animals [11].

### Determination of IL-1β level in joint exudates

Joint exudates were used to determine IL-1β levels following the instructions of the manufacturer of a commercially available IL-1β ELISA kit (R&D Systems, São Paulo, SP, Brazil).

### Immunohistochemistry

Synovial membranes were used to assess the immunoexpression of the nuclear factor kappa B (NF-κB p65) and tumor necrosis factor (TNF)-α, using commercially available kits (Abcam, Cambridge, United Kingdom). Briefly, after deparaffinization, tissue sections were incubated with hydrogen peroxide and washed. After incubation in citrate buffer, specimens were subjected to slight heating in a microwave oven. After overnight unspecific blocking with rabbit serum, the samples were incubated with the rabbit anti-NFκB or anti-TNFα antibody (diluted 1:200 in PBS plus 1% of BSA, 2 h). After rinsing, the sections were incubated with a secondary biotinylated anti-rabbit IgG antibody. The reaction product was detected using Envision™ System-HRP (AEC) complex (DAKO, Carpinteria, CA, USA), and the color of the reaction was developed with diamino-benzidine tetrahydrochloride (DAKO, Carpinteria, CA, USA). The slides were counterstained with Mayer’s hematoxylin. The intensity of the staining was analyzed under light microscopy using Image J™ software [12].

### Statistical analysis

Data were analyzed using GraphPad Prism 6 (GraphPad Software, Inc., San Diego, CA, USA). Results are reported as means ± standard deviation (SD) or medians (range), as appropriate. Student’s “t” test, one-way ANOVA followed by Tukey’s test or Kruskal-Wallys were used to compare differences between means and medians, as appropriate.

## Results

### MgCl_2_ reduces cell infiltration, hypernociception, and IL-1 release in acute zymosan arthritis

**Fig. 1:**
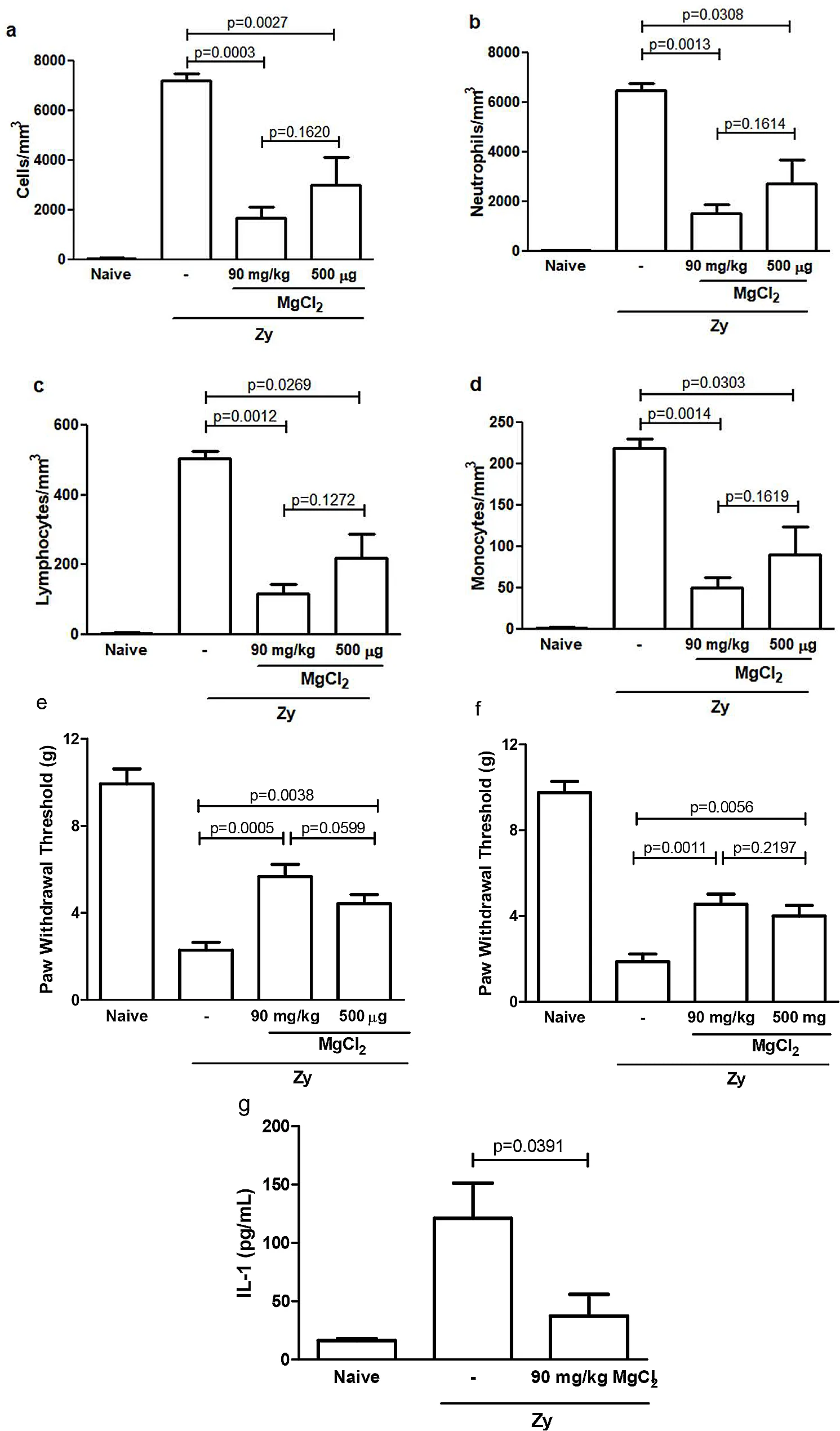
Mice that received 0.1 mg Zymosan (Zy) intra-articular (i.art.) or saline were treated with 500 µg MgCl_2_ i.art. or 90 mg/kg MgCl_2_*per os*. Cell counts (a-d) in joint exudates 6 h after Zy injection. Hhypernociception (paw withdrawal threshold) measured with von Frey filaments at 3 h (1e) and 5 h (1f). Interleukin(IL)-1 level in joint exudates 6 h after Zy injection (1 g). Data are mean ± (SD) (n = 6/group) compared using one-way ANOVA followed by Tukey’s test

As shown in Fig. 1a, Zy injection induced a prominent leukocyte migration, with predominance of polymorphonuclear cells (> 85%), into the knee joint after 6 h; pretreatment via systemic (*p* = 0.0003) or local (intra-articular) (*p* = 0.0027) administration of MgCl_2_ promoted a remarkable and highly significant reduction of cell infiltration, as shown in Fig. 1a. The reduction in cell counts was mostly of PMN, although numbers of lymphocytes and monocytes were also reduced (Fig. 1b-d). Interestingly, though not reaching statistical significance, reduction of cell counts appeared more pronounced after systemic (oral) administration of MgCl_2_ as compared to the intra-articular injection local administration (Fig. 1a-d).

Regarding pain, the hypernociceptive response provoked by injection of Zy into the mouse joints was significantly reduced by both systemic and local administration of MgCl_2_ solutions, happening as early as 3 h following Zy injection, being sustained until 5 h (Fig. 1d-e). The antinociceptive response was more pronounced in mice that received systemic MgCl_2_ as compared to local administration (Fig. 1e).

Given the increased reduction in cell infiltration and hypernociception following systemic administration of MgCl2, further studies were done using this dosage. Reduction of the release of the inflammatory cytokine IL-1β into joint exudates is seen in animals that received MgCl_2_ systemically, as compared to animals treated with vehicle (saline) (Fig. 1g).

### Histopathological evaluation

As expected, injection of Zy induced a significant increase in cell infiltration into the synovia as well as synovial hyperplasia. Such changes were significantly reduced after treatment of the mice with the MgCl_2_ solution regardless of being a systemic or local administration (Table 1).

**Table 1:** Synovial histology of zymosan arthritis following MgCl_2_ administration

Group	Cell Infiltration	Synovial Proliferation
**Naïve**	0 (0.0–0.0)	0 (0.0–1.0)
**Control**	2.0* (2.0–3.0)	1.0* (1.0–3.0)
**MgCl (p.o.)**	1.5* (1.0–2.0)	0.5^**#**^ (0.0–1.0)
**MgCl (i.a.)**	2.0* (1.0–2.0)	0.5 (0.0–1.0)

Mice (n = 6/ group) received (90 mg/kg p.o or 500 µg intra-articular, i.a.) or saline i.a. (control) 30 min prior to 0.1 mg/25µL zymosan i.a. Naive mice received saline. Animals were killed under anestesia at 6 h. Results represent synovial histopathology scores (median; range). * *p* < 0.05 compared to naive and # *p* < 0.05 compared to control

### **MgCl**_**2**_**decreases immunoexpression of NFkB and TNFα in the Synovia.**

**Fig. 2:**
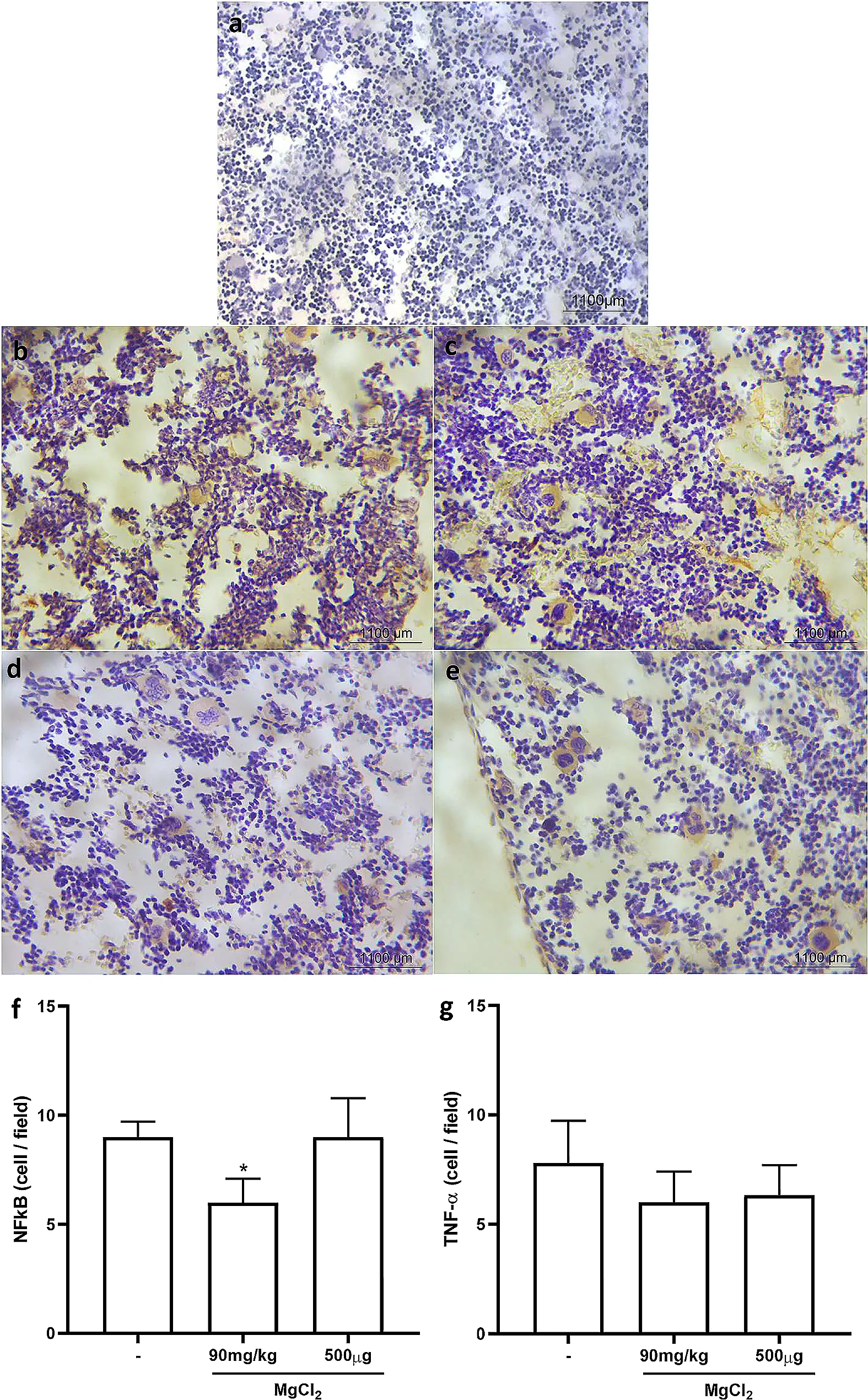
Mice that received intra-articular (i.art.) 0.1 mg Zymosan (Zy) or saline into joints were treated with 90 mg/kg MgCl_2_*per os*. Synovia collected 6 h after Zy injection were processed for immunohistochemistry (see text for details). Representative illustration and semiquantitative data of the immunoexpression of NFκB (b,d,f) and TNF-(c,e,g) as compared to control (a). Data are mean ± (SD) (n = 6/group) compared using Student’s “t” test (Original magnification x40)

Figure 2 illustrates photomicrographs of the immunostaining for analysis of the expression of NFκB and TNFα in the synovia of mice subjected to Zy arthritis treated with a MgCl_2_ solution. There is intense immunostaining for both NFκB (2b) and TNFα (2c) in mice that received Zy and saline, as compared to naïve mice (2a). Systemic pretreatment with MgCl_2_ significantly reduced immunostaining for NFκB (2d, 2f). Immunoexpression of TNFα was also partially reduced after systemic MgCl_2_ administration, though not reaching statistical significance (2e, 2g).

## Discussion

Our present data reveal that MgCl_2_ solutions, given systemically (*per os*) or locally (intra-articularly), significantly decreased acute inflammatory cell migration and the hypernociceptive response in a classic and severe acute arthritis model. This effect was associated with a reduced expression of NFκB, an intracellular protein complex linked to transcription of inflammatory cytokines, including IL-1 and TNF [13]. The fact that our treatment strategy, being prophylactic, prevented inflammation from developing at a very early stage, both when injected systemically or locally, suggests that resident cells of the synovium were probably affected by MgCl_2_ administration. Indeed, inflammatory migrating cells in zymosan arthritis are seen circa 3 h following Zy administration. Given that mice treated with MgCl_2_ had significant pain relief as early as 3 h post Zy injection, it might well be that MgCl_2_ was acting in local, resident synoviocytes, prior to the peak of cell infiltration in this arthritis model [14]. The fact that MgCl_2_ was effective when injected locally does also suggest that an anti-inflammatory activity via interfering with the activation of resident cells is likely.

Previous studies associated low serum Mg levels with increased severity of rheumatoid arthritis [15]. Indeed, using data from the National Health and Nutrition Examination Survey (NHANES) cohort, women with lower dietary Mg intake had decreased prevalence of rheumatoid arthritis, pointing to a possible protective effect of this micronutrient in inflammatory arthropathies [16]. We have also alluded above to an increased prevalence of CPPD in individuals with Mg deficiency [5]. Lower serum Mg levels were also associated with increased severity in OA patients [6]. In the metabolic context, higher dietary Mg intake was associated with a lower risk fracture associated with osteoporosis [17]. In conjunction, these data suggest a beneficial effect of either the administration of Mg or at least maintenance of appropriate serum Mg levels in patients with musculoskeletal diseases.

Regarding antinociception (pain relief), it was shown that Mg reduces pain in arthritis patients and intra-articular administration of Mg reduces postoperative pain in patients undergoing knee arthroscopy [18, 19]. The analgesia provided by Mg was linked to the activation of *N*-methyl-D-aspartate (NMDA) receptors [3]. Our results advanced in proposing possible mechanisms for the analgesic and anti-inflammatory activity of MgCl_2_ administration, which would be due to reduction of the release of inflammatory cytokines, namely IL-1β and possibly also TNF-α. These are potent inflammatory mediators linked to pain mechanisms and joint damage in various arthritis models [13]. Actually, inhibition of TNF by using monoclonal antibodies or a soluble TNF receptor are currently used to treat rheumatoid arthritis, spondyloarthropathies, and psoriatic arthritis promoting pain relief and halting joint damage in some patients [20, 21]. Regarding IL-1β, a *post-hoc* analysis of a clinical trial showed that patients with cardiovascular disease secondary to atherosclerosis treated with canakinumab, an anti-IL-1β antibody, had a reduced risk of undergoing hip or knee arthroplasty secondary to OA [22]. The decreased release of IL-1β that we obtained following MgCl_2_ administration was associated with reduced expression of NF-κB in the inflamed synovium, thus revealing a possible intra-cellular mechanism of action. In keeping with this assumption, it was shown that exposure of chondrocytes from OA patients with Mg incorporated into chondroitin sulfate increased cell viability and inhibited apoptosis, while being associated with reduction of the expression of inflammation-related genes. Additionally, MgSO_4_ administration was shown to reduce IL-1 release and mRNA expression for IL-1 in synovial tissue in an animal model of OA [3, 23]. We may speculate that exogenous administration of magnesium, as done in our study, leads to suppression of the activation of NF-kB, thus decreasing the release of inflammatory mediators including IL-1β. Indeed, it was shown that increased Mg levels decrease in vitro cytokine production by macrophages and higher Mg levels were associated to lower levels of inflammatory cytokines during arthritis [24]. Also, magnesium-based scaffolds were shown to promote chondrogenesis through controlled Mg^2+^ release eliminating the destructive effect of activated macrophages on chondrocytes [25]. Moreover, using an inflammatory model, splenocytes collected from mice subjected to a high content Mg diet were shown to produce more IL-10, a cytokine linked to anti-inflammatory activity, as compared to animals exposed to a diet with lower Mg content. This was associated to alterations of the intestinal microbiota, with the participation of FOXp3 Treg cells. Besides adding another mechanism of Mg role in inflammation, the fact that those results were achieved with oral supplementation of Mg opens the possibility of testing clinical translation to humans [26]. Other mechanisms may operate to explain the anti-inflammatory activity of Mg. Previous in vitro and in vivo experimental studies associate low Mg levels with increased release of IL-1, IL-6, and TNF-α by neutrophils and macrophages. Low Mg levels were also shown to trigger the release of pro-inflammatory cytokines following activation of NF-κB in endothelial cells, while also upregulating the expression of adhesion molecules in the endothelium, thus facilitating migration of inflammatory cells into inflamed tissues [27]. Mg ions do also display antioxidant activity with low Mg levels being associated with increased production of reactive oxygen species by neutrophils [7, 8]. Additionally, by operating as a calcium (Ca) antagonist, Mg blocks Ca access to Ca-binding proteins, leading to mast cell degranulation and activation of adhesion molecules, thus promoting inflammation [28, 29].

Protein phosphatase magnesium dependent 1 A (PPM1A) is involved in inflammation and immune responses at least partially by regulation of transforming growth factor-β (TGF-β) signalling via phosphorylation of SMAD2. It was also shown that PPM1A levels are increased in synovial fluid of patients with rheumatoid arthritis. IL-1β and TNF, which are produced following activation of NF-κB, are inducers of PPM1A production in chondrocytes. A recent study has shown increased expression of PPM1A in chondrocytes, but not synoviocytes, of mice subjected to experimental OA. Furthermore, genetic ablation of PPM1A or pharmacological inhibition of its enzyme activity protected joint damage in mice subjected to an OA model, with decreased expression of IL-1β and reduced activity of metalloproteinases [29]. We may speculate that our findings of decreased IL-1β levels and reduced NF-κB expression following Mg administration result in decreased PPM1A activation, thereby reducing inflammation.

Our study is not without limitations. Although in vivo, we used an experimental arthritis model and our results have to be reproduced in humans. Other important mediators, including prostanoids, which are associated with pain development in arthritis, were not investigated. It will also be important to uncover mechanisms of action using isolated experiments with resident joint cells, such as synoviocytes and chondrocytes, as well as with inflammatory cells that migrate to joints.

## Conclusion

In summary, we demonstrate that systemic or local administration of MgCl_2_ has anti-inflammatory activity in an acute arthritis model, which is associated to decreased expression of NF-κB and reduction of inflammatory cytokine release. MgCl_2_ has been used in lay medicine to treat musculoskeletal pain, particularly secondary to OA. Our data add relevant information regarding the efficacy of this safe compound. The use of nutraceuticals and phytocompounds is a very frequent initiative of automedication of patients seeking health benefit. Our results suggest that designing prospective well-designed protocols to address efficacy and safety of MgCl_2_ preparations in patients with chronic arthritis is warranted.

## Data Availability

All data generated or analysed during this study are included in this published article.

## Abbreviations

HE: Hematoxylin-eosin
IL-1: Interleukin-1
i.art.: Intra-articular
NMDA: N-methyl-D-aspartate
NFκB: Nuclear factor kappa B
OA: Osteoarthritis
(TNF)-α: Tumor necrosis factor
Zy: Zymosan
